# Sex differences in endothelial function of aged hypertriglyceridemic rats – effect of atorvastatin treatment

**DOI:** 10.2478/v10102-012-0025-2

**Published:** 2012-09

**Authors:** Ruzena Sotnikova, Barbora Bacova, Jana Vlkovicova, Jana Navarova, Narcis Tribulova

**Affiliations:** 1 Institute of Experimental Pharmacology & Toxicology, Slovak Academy of Sciences, Dubravska 9, 84104 Bratislava, Slovak Republic; 2 Institute for Heart Research, Slovak Academy of Sciences, Dubravska 9, 840 05 Bratislava, Slovak Republic

**Keywords:** aged hypertriglyceridemic rats, sex differences, endothelial function

## Abstract

The aim of the study was to test the hypothesis that the effect of atorvastatin on endothelium-dependent relaxation of the superior mesenteric artery (SMA) may differ in male *vs.* female aged hypertriglyceridemic rats (HTGs). Experiments were performed on 11-month-old male and female Prague hereditary HTGs. Atorvastatin (ATO) was administered *p.o.* in the dose of 0.30 mg/100g/day. Controls received vehiculum. After two months of ATO administration blood pressure, serum triglycerides (TG) and total cholesterol (CHOL) were determined. Endothelial function of SMA was studied *in vitro* using evaluation of relaxant responses of precontracted SMA to acetylcholine. The serum TG of control male HTGs were found to be statistically higher than those of female controls, while CHOL and blood pressure did not share gender differences. Responses of SMA of female control HTGs were statistically decreased compared to their male counterparts. ATO treatment induced decrease in blood pressure and TG of both males and females, yet CHOL values were reduced only in females. The protective effect of ATO on SMA endothelial function was much more pronounced in females compared to males.

We conclude that vascular endothelial dysfunction of aged HTG rats is more severe and more attenuated by ATO in females compared to males. The protective effect of ATO on vascular endothelial function does not seem to depend solely on its lipid lowering action.

## Introduction

Hypertriglyceridemia participates in the development of atherosclerosis and hypertension in humans (Gillespie *et al.,* 2012; Gupta *et al.,* 2012). Statins are most widely prescribed drugs in the treatment of dyslipidemia. The 3-hydroxy-3-methylglutaryl coenzyme A (HMGCoA) reductase inhibitors, statins, lower plasma cholesterol levels and have been associated with reduced morbidity and mortality in patients with coronary artery disease (Scandinavian Simvastatin Survival Study, 1994; Sacks *et al.*, 1996). Statins are presumed to cause regression or stabilization of atherosclerotic plaques by lowering serum cholesterol levels (Libby, 1995). However, the beneficial effects of statins on coronary artery disease are not limited only to their ability to lower plasma cholesterol (Scandinavian Simvastatin Survival Study, 1994) but they involve various pleiotropic effects on atherosclerosis, *e.g.* anti-inflammatory effects, reduction of plaque thrombogenicity, inhibition of cellular proliferation and migration, and improvement of endothelial function (Vaughan *et al*., 2000). Endothelial injury and dysfunction is an important initial event in atherogenesis and restenosis (Celermajer, 1997; Xi *et al*., 2007; Sima *et al*., 2009), while inflammation significantly contributes to endothelial activation and subsequent adhesion of polymorphonuclear leukocytes to vascular endothelium (Mazzone *et al*., 1993; Lush *et al*.*,* 2000). Thus pleiotropic effects of statins may enhance the outcome in patients with cardiovascular diseases. Statins have been widely prescribed medications yet in their therapeutic response considerable variability still remains. At present, little is known about the impact of gender on lipid lowering as well pleiotropic effects of atorvastatin. Nevertheless, there are gender-related differences in the propensity to cardiovascular diseases (Mounier-Vehier *et al.,* 2012). Thus, the aim of the study was to test our hypothesis that the effect of atorvastatin (ATO) on endothelium-dependent relaxation of the superior mesenteric artery (SMA) may differ in male *vs* female aged hypertriglyceridemic (HTG) rats.

## Methods

Experiments were performed on 11-month-old male and female Prague hereditary hypertriglyceridemic rats (HTGs). The rats were maintained under a 12 h light/dark cycle with free access to water and a standard laboratory diet. Animal housing, care and experimental procedures were conducted under the guidelines of the Animal Ethics Committee and were approved by the State Veterinary and Food Administration of the Slovak Republic.

Atorvastatin (Zentiva, Slovakia) was administered *p.o*. for two months in the dose of 0.30 mg/100g/day. Control rats (C) received vehiculum. At the end of the experiment (weight of animals was 373.6±18.5 g), blood pressure and plasma lipids were recorded and endothelial function of SMA was tested *in vitro* under isometric conditions.

Blood pressure was measured noninvasively by tail-cuff plethysmography using the Statham pressure transducer P23 XL (Germany). Blood was collected at the end of experiment and used for triglycerides (TG) and cholesterol (CHOL) assay, using a commercial kit from Biolab Diagnostics.

The superior mesenteric artery (SMA) was removed from male and female HTG rats and immersed in physiological salt solution (PSS). Adherent tissues were removed and appr. 2 mm long rings were cut. Care was taken not to damage the endothelium. The rings were mounted between two platinum hooks. The tissue chamber contained PSS (in mmol/l): NaCl 112.0, KCl 5.0, KH_2_PO_4_ 1.0, MgCl_2_ 1.2, CaCl_2_ 2.5, NaHCO_3_ 25.0 and glucose 1.5. PSS was bubbled with a mixture of 95% O_2_ and 5% CO_2_ (pH=7.4) at 37^o^C. The preparations were connected to an isometric transducer (Experimetria Hungary), stretched passively to the resting tension of 15 mN and allowed to equilibrate for 1 hr. We evaluated the response of phenylephrine (1µmol/l)-precontracted rings to acetylcholine (ACh) in cumulative concetrations (10 nmol/l – 10 µmol/l) before and after NO synthase inhibition with NG-nitro-L-arginine methyl ester (NO-resistant relaxation).

Values of pD_2_ (logarithm of IC_50_ which represents the concentration of a drug required for 50% inhibition *in vitro*) were calculated on using GraphPad Prism. Results were compared by two-way ANOVA with Bonferroni test.

## Results

In our experiments the serum TG of control aged male HTGs were statistically higher than those of female controls. However we did not find gender differences in serum total cholesterol (CHOL) or blood pressure. ATO treatment decreased blood pressure as well as serum TG of both males and females, while serum CHOL only in females (Table 1).

**Table 1:** Effect of atorvastatin (ATO) on serum triglycerid (TG in mmol/l), serum total cholesterol (CHOL in mmo/l) and blood pressure in mm Hg.

Group		TG (mmol/l)	CHOL (mmol/l)	Blood pressure (mm Hg)
Male	Control	2.67±0.12	1.61±0.13	134.30±6.61
	ATO treated	1.39±0.11**	1.82±0.27	115.70±1.51**
Female	Control	1.63±0.09^xx^	1.34±0.03	133.82±4.21
	ATO treated	1.10±0.09**	1.03±0.08*	120.32±2.32*

* *p*<0.05 *versus* corresponding control

** *p*<0.01 *versus* corresponding control

xx *p*<0.01 *versus* male rats

**Figure 1:**
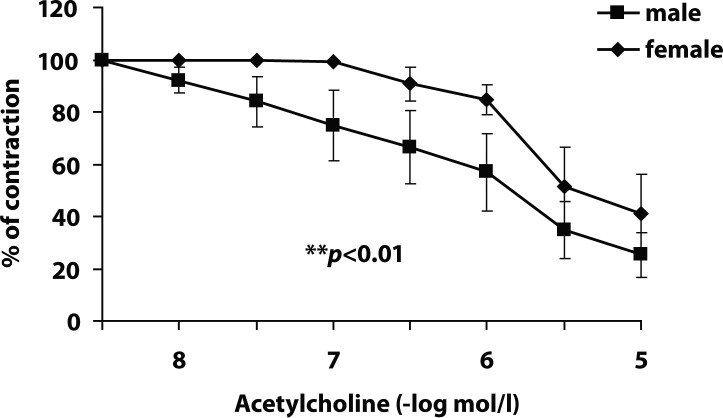
Effect of acetylcholine on phenylephrine (1×10^−6^mol/l)- precontracted superior mesenteric artery. Results are expressed in % of precontraction. Data are means ± S.E.M. of 8 experiments. **p<0.01 – difference between male and female.

**Figure 2:**
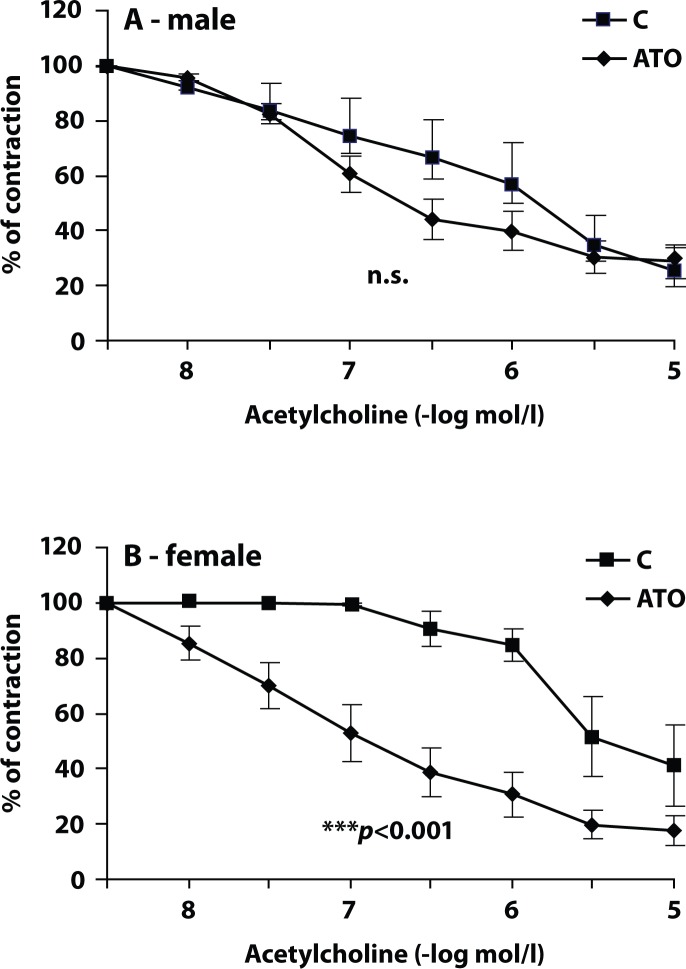
Effect of atorvastatin (ATO) treatment on responses of phenylephrine (1×10^−6^mol/l)-precontracted male and female superior mesenteric artery to acetylcholine. C – controls. A – males, B – females. Results are expressed in % of precontraction. Data are means ± S.E.M. of 8 experiments. ***p<0.001 compared to corresponding control; n.s. – non-significant difference.

Responses of SMA to acetylcholine were significantly less pronounced in female rats (pD_2_ 6.48±0.36) compared to male rats (pD_2_ 7.04± 0.34) (Figure 1). Administration of ATO had no significant effect on acetylcholine-induced relaxation of male SMA. The pD_2_ value was 7.60±0.08 (Figure 2A). The SMA of female rats treated with ATO responded to acetylcholine with improved relaxation compared to untreated animals (pD_2_ 7.65±0.10, Figure 2B). Even on comparing endothelial relaxations of males and females after ATO-treatment, those of females were statistically more pronounced (*p*<0.01).

The NO-resistant part of endothelium-dependent relaxation of SMA was not influenced either by the gender of animals or by treatment (*p*>0.05).

## Discussion

Our study showed sex differences in endothelial function and ATO efficacy in aged hypertriglyceridemic rats. We found that responses of SMA to acetylcholine of male controls were more pronounced compared to those of female controls. It is known that estrogens have a beneficial effect on vascular endothelial function (Liebermann *et al*., 1994) and enhance basal nitric oxide release (Sudhir *et al*., 1996), yet in aged females this protective effect of estrogens is blunted. Aging counteracts the protective role of estrogen related vascular responses, including depletion of bone marrow derived endothelial progenitor cells required for vascular repair and vascular function (Pepine *et al*.*,* 2006). Liu *et al.* (2001) found that plasma concentrations of 17β-estradiol in female rats decreased with age. As we used aged females in our experiments, the protective effect of estrogens on endothelium-dependent relaxation was probably dampened, as reflected in a depressed response of SMA to acetylcholine in comparison to male SMA.

On evaluating the lipid profile of animals, we found higher concentrations of TG in serum of control male HTGs compared to females. The study of Pitsavos et al. (2008) showed in human familial hyperlipidemia higher TG levels and higher cardiovascular risk in males compared to their female counterparts. In our experiments, we also found increased levels of TG, however in blood pressure we did not find a significant sex difference. Even the endothelium-dependent relaxation of male SMA was more pronounced compared to female rats. Banos *et al*. (2005) referred higher activities of the antioxidant enzymes catalase and glutathione peroxidase in male HTGs, which may be, at least in part, responsible for the results found in male HTGs in our experiments.

Further we investigated effects of the most frequently used statin – atorvastatin on endothelial function of SMA of HTGs in relation to gender. We also studied the effect of ATO administration on blood pressure, serum TG and CHOL. In our experiments, administration of ATO to rats had a beneficial effect on blood pressure and TG of both sexes. Comparably, the results of Bacova *et al.* (2010) showed atorvastatin to improve these parameters in 5-month-old hypertriglyceridemic male rats. Blood CHOL, however, was statistically improved only in female rats in our experiments. Further we found a more intensive protective effect of ATO on SMA endothelial function in females.

Clinical trials reported that both women and men derived benefit from intensive atorvastatin therapy after acute coronary syndrome (Truong *et al*., 2011). Thus the authors have suggested that sex should not be a factor in determining intensive statin therapy. Nevertheless, Sakabe *et al.* (2008) found gender differences in some lipid alterations, including products of lipid peroxidation – thiobarbituric acid reactive substances and small, dense LDL-cholesterol concentrations after 3-month atorvastatin therapy, and these were greater in women. We can thus speculate that the more intensive ATO treatment benefit on SMA endothelium established in our female rats may be a consequence of ATO effect on parameters of oxidative stress.

On the basis of our results we conclude that vascular endothelial function is more depressed and better recovered by ATO in aged female HTG rats compared to males. The protective effect of ATO on vascular endothelial function does not appear to depend solely on its lipid lowering action. These findings should be taken into account in clinical conditions.
